# Ketogenic metabolic therapy: low-carbohydrate interventions as novel neuroprotective strategies for cognitive dysfunction in diabetes

**DOI:** 10.3389/fnagi.2026.1777834

**Published:** 2026-04-24

**Authors:** Ying Xun, Wenqing Zeng

**Affiliations:** 1 Department of Endocrinology, Tongde Hospital of Zhejiang Province, Hangzhou, China; 2 Department of Intensive Care Medicine, The Second Affiliated Hospital of Zhejiang Chinese Medical University, Hangzhou, China

**Keywords:** cognitive dysfunction, diabetes mellitus, gut-brain axis, LCKDs, low-carbohydrate ketogenic diet, mild cognitive impairment, nutritional ketosis

## Abstract

Cognitive dysfunction is an increasingly recognized complication of diabetes, contributing substantially to morbidity in the aging population, yet disease-modifying therapies remain scarce. Dietary intervention, a cornerstone of diabetes management, may offer neuroprotective potential. Low-carbohydrate ketogenic diets (LCKDs), typically restricting carbohydrates to < 50 g/day, effectively improve glycemic control and metabolic health. Emerging preclinical and clinical evidence suggests that LCKDs may also confer cognitive benefits through mechanisms including enhanced mitochondrial bioenergetics, reduced neuro-inflammation, and modulation of the gut-brain axis. This narrative review critically evaluates the current evidence regarding the efficacy, mechanisms, and safety of LCKDs for managing diabetes-associated cognitive dysfunction, identifies key limitations in the existing literature, and proposes a framework for future research to enhance translational value.

## 1 Introduction

The global prevalence of diabetes, particularly type 2 diabetes mellitus (T2DM), continues to rise, driven largely by population aging and the obesity epidemic (GBD 2021 Diabetes Collaborators, 2023; Sun et al., 2022; Antza et al., 2021; Lauder et al., 2023). This escalating trend contributes to a growing burden of diabetic complications on healthcare systems worldwide. Common macrovascular and microvascular complications, such as cardiovascular disease, nephropathy, and retinopathy, have long been the focus of clinical attention (Luk et al., 2025; Iwasaki et al., 2025; Alrubaiee, 2025; Nokihara et al., 2025). In recent years, however, cognitive dysfunction has emerged as a significant and increasingly recognized complication of diabetes. Epidemiological evidence indicates that among individuals aged 60 years and older with T2DM, the prevalence of dementia approaches 20% (Srikanth et al., 2020). Compared with normoglycemic counterparts, this population exhibits a 1.44-fold increased risk of mild cognitive impairment (MCI) (Jia et al., 2020) and a 2.8-fold higher risk of dementia (Schnaider Beeri et al., 2004). These findings highlight the pressing need for effective strategies to prevent and treat cognitive decline in diabetic patients, an area that remains largely unmet and warrants urgent investigation.

Despite growing recognition of this clinical challenge, several critical gaps persist in the existing literature. First, the pathophysiological mechanisms linking diabetes to cognitive impairment-encompassing insulin resistance, chronic neuro-inflammation, mitochondrial dysfunction, and vascular damage-are complex and incompletely understood, complicating the development of targeted interventions. Second, while dietary approaches have shown promise, most evidence derives from preclinical models or studies in non-diabetic populations with mild cognitive impairment or Alzheimer's disease, limiting direct applicability to diabetic cohorts. Third, available human studies are characterized by substantial heterogeneity in intervention protocols-including variations in carbohydrate thresholds, dietary fat composition, ketosis monitoring, and duration-as well as inconsistent outcome measures, which hinder cross-study comparison and synthesis. Fourth, safety considerations specific to diabetic patients, particularly regarding medication interactions (e.g., insulin, sodium-glucose cotransporter 2 inhibitors), renal function, and long-term metabolic effects, remain inadequately addressed. These gaps underscore the need for a critical evaluation of current evidence and a structured framework to guide future research.

Current management approaches for cognitive dysfunction in diabetes encompass multifactorial risk factor modification, treatment of underlying pathologies, and symptomatic interventions (Yang et al., 2022b; Sjöholm et al., 2025; Liu et al., 2020; Xu et al., 2025). Dietary patterns are increasingly recognized as modifiable risk factors, with healthy diets holding significant potential for neuroprotection. The low-carbohydrate ketogenic diet (LCKD), historically used for epilepsy, has demonstrated efficacy in improving hyperglycemia, facilitating weight loss, and ameliorating metabolic dysregulation in diabetes (Bruci et al., 2020; Ren et al., 2024; Merovci et al., 2024; Leung et al., 2025). Emerging evidence suggests that nutritional ketosis, characterized by beta-hydroxybutyrate (BHB) concentrations typically ranging from 0.5 to 5 mmol/L, confers dual benefits: it optimizes systemic metabolism and directly supports brain health by providing an alternative fuel source (Lennerz et al., 2021; Tosefsky et al., 2026). This review compiles and evaluates the evidence for LCKDs as a neuroprotective intervention for cognitive dysfunction in diabetes, with a focus on mechanistic pathways, clinical evidence, intervention heterogeneity, and safety considerations.

## 2 Defining the intervention and the target condition

### 2.1 Low-carbohydrate ketogenic diets: a spectrum of interventions

Dietary regimens restricting carbohydrates are heterogeneous. A daily carbohydrate intake below 130 g is generally classified as low-carbohydrate, while intakes ≤ 50 g per day is typically required to induce and maintain nutritional ketosis, defining a ketogenic diet (Charlot and Zoll, 2022; Sakr et al., 2023). Critically, not all low-carbohydrate diets are ketogenic. Moreover, ketogenic diets themselves vary considerably in their macronutrient composition. These include classic low-carbohydrate ketogenic diets (LCKDs), which are high in fat and moderate in protein; modified Mediterranean-ketogenic diets (MMKDs); and very-low-calorie ketogenic diets (VLCKDs) (Sukkar and Muscaritoli, 2021; Di Renzo et al., 2023; Calcaterra et al., 2023). These variations in macronutrient composition, calorie prescription, and duration significantly influence ketosis depth, metabolic effects, and tolerability, necessitating careful consideration when interpreting study outcomes.

### 2.2 Diabetes-associated cognitive dysfunction: phenotypes and outcomes

Cognitive dysfunction in diabetes represents a multifactorial continuum, typically classified into asymptomatic decline, MCI, and dementia. The associated dementia phenotypes are broadly categorized into two types: vascular dementia (VaD), resulting from cerebrovascular damage, and Alzheimer's disease (AD)-like pathology, driven by neurodegenerative alterations potentially accelerated by diabetes (Li et al., 2023; van Sloten et al., 2020; Zhang et al., 2023). Key cognitive domains affected include information processing speed, executive function, and memory (Murali et al., 2025; Palta et al., 2014), with assessment relying on standardized neuropsychological tools such as the Montreal Cognitive Assessment (MoCA), Mini-Mental State Examination (MMSE), and domain-specific tests (Wang and Chen, 2025; Wei et al., 2025; Carlew et al., 2021). Interpretation of intervention effects requires simultaneous consideration of diabetes-related parameters, including hypoglycemia history (Lee et al., 2014), microvascular burden (Satapathy et al., 2025), and neuroimaging markers like white matter hyperintensities (WMH) (Lee et al., 2014; Sugimoto et al., 2025) and cerebral blood flow (CBF) (Li et al., 2024; Mondal et al., 2024; Chen et al., 2025). To structure the evidence, Table 1 summarizes the key cognitive outcomes and relevant diabetes-related parameters reported in clinical studies.

**Table 1:** Summary of key outcomes in studies of ketogenic diets for cognitive dysfunction.

Author/Year/[reference]	Diabetes or not	Types of animal/participants	Data of laboratory/ clinical	Types of ketogenic diets	Neurological assessment tools	Relevant diabetes/CNS parameters	Effect (conclusion)
(Tosefsky et al. 2026)	Not	Participants	Clinical	MeDi-MCT vs. MeDi-KD	MDS-UPDRS, MoCA, PD-CRS	BHB, Brain BHB, plasma medium chain fatty acids, MDS-UPDRS	MeDi-MCT(Positive) MeDi-KD(Negative)
(Luong et al. 2025a)	Not	Participants	Clinical	KD	Neuropsychological test	HbA1c, BHB, BDNF, CBF	Positive
(Patoine et al. 2025)	Prediabetic adults	Participants	Clinical	MMKD MD	MoCA	BMI/DCCS, FName, Flanker, LSWM, PSMT, RAVLD, rey auditory verbal Learning Delay, Speed Matching Test	Obesity (Positive); Not-obesity: (Negative)
(Roslund et al. 2025)	Not	Middle-aged female mice	Laboratory	KD	Not mentioned	BHB, glucose catabolism, glucose-alanine cycle, brain glucose-dependent energy metabolism, lactate production downstream from glucose/hippocampus metabolome, cortex metabolome, hippocampal metabolites involved in myelinogenesis	Positive
Arabacı Tamer et al. (2025)	Diabetes	Adult male Wistar rats	Laboratory	KD	Hole-board test, Elevated plus maze test, Object recognition test	MDA, GSH, S100β, TNF-α, IL-6, BDNF, 8-OHdG, Ketone levels, Fasting glucose levels/Neuronal integrity (histopathological and immune-histochemical analyses of CA1, CA3, and dentate gyrus regions)	Positive
Abdel-Kareem et al. (2025)	Insulin-resistant	Rats	Laboratory	KD	MWM	IR index (homeostasis model assessment), BDNF, IDE activity, GSK3β activity, Tau protein, Aβ	Negative
(Park et al. 2024)	Not	Mice	Laboratory	MMKD	Neurocognitive and neuromuscular function	GTT, ITT, object Recognition Test, location Memory Test, T-maze Spontaneous Test, neuromuscular and motor function test	Positive
(Ren et al. 2024)	Diabetes	db/db mice	Laboratory	KD	Not mentioned	Fasting blood glucose, body weights/mRNA-seq analysis of brain gene expressions, DEGs in the brain, GSEA Proteasomal activity in the brain	Positive
Acuña-Catalán et al. (2024)	Not	aged mice	Laboratory	KD	Elevated plus maze, Novel object recognition, Open field, Y-maze, Barnes maze, Rotarod	Glycemia, BHB blood concentration/Elevated plus maze, open field, Y-maze, Barnes maze, Rotarod, novel object recognition, hippocampal slices electrophysiology, golgi staining, morphometric analysis of pyramidal neurons	Positive
(Di Lucente et al. 2024)	Not	Aged C57BL/6 mice	Laboratory	KD	Open field, Y-maze, Barnes maze	BHB/LTP, BDNF, p-ERK, p-CREB, glutamate release	Positive
(Gureev et al. 2024)	Not	Mice	Laboratory	BHB	MWM, String Test	String test, Rod test, Rotarod test, MWM, Mitochondrial metabolism, Tissue ATP levels, Nrf2/ARE signaling pathway activity, Antioxidant defenses, Inflammation status	Positive
(Jiang et al. 2023)	Not	APP/PS1 mice	Laboratory	KD	NORT,Y-maze spontaneous alternation test	Blood sugar levels, hyperinsulinemia, glucose metabolism, insulin sensitivity/Recognition index, Y-maze spontaneous alternation, Aβ deposition, GFAP expression, Iba1 expression, Nrf2 protein level HO-1 protein level, Phospho-IκBα protein level, NF-κB p65 protein level, Learning and memory capacity	Positive
(Rutkowsky et al. 2024)	Not	TgF344-AD rats	Laboratory	KD	Elevated plus maze, open field, grip strength, rotarod, Barnes maze	Glucose, ketone measurements/Barnes maze performance, rotarod test performance, grip strength, Aβ40, Aβ42, pTau, total Tau	Negative
(Dilmore et al. 2023)	Prediabetic adults	participants	Clinical	MMKD	Not mentioned	Hb1Ac,insulin/Aβ42, tau, cerebral ketone body uptake, cerebral perfusion	Positive
(Qin et al. 2023)	Not	APP/PS1mice	Laboratory	KD	MWM,Y maze	BHB/learning and memory ability, Brain iron deposition, Aβ, Neuronal ferroptosis, Nrf2-mediated ferroptosis pathway activity	Positive
(Di Renzo et al. 2023)	Not	Participants	Clinical	MMKD	Fibromyalgia Tests	EQ-5D	Positive
(Yang et al. 2022a)	Not	Female C57BL/6 mice	Laboratory	KD	MWM	BHB, Blood glucose/Aβ, p-tau, BACE1, BDNF, DCX, Iron deposition, Lipid peroxides accumulation, MDA, GSH, GPX4	Positive
(Kumar et al. 2022)	Not	Participants	Clinical	MMKD vs. AHAD	ADNI-2, NIA-AA, LP, MRI	Aβ1-42, p181-tau, NfL, Ng, glutamate, glutamate receptor ionotropic NMDA1, glutamate receptor ionotropic NMDA2A, glutamate receptor ionotropic NMDA2B, glutamate receptor ionotropic AMPA type subunit 1, AGEs, MCT2	Positive
(Brinkley et al. 2022)	Not	Participants	Clinical	MMKD vs. AHAD	ADNI-2, NIA-AA	Aβ42, Aβ40, total tau, tau-p181, Ng, sTREM2, YKL40, NfL, AChE activity, BChE activity, Aβ42/Aβ40 ratio, Aβ42/tau-p181 ratio, tau-p181/tau ratio, AChE/BChE activity	Positive
(Wang et al. 2021)	Not	PTZ-induced and kindled rats	Laboratory	KD	MWM	GluR1 (mRNA and protein expression), NR2B (mRNA and protein expression), MAPK activity, CAMKII activity	Positive
(Neth et al. 2020)	Not	Participants	Clinical	MMKD	FCSRT, WMS-R, ADAS-Cog12	HbA1c, Glucose, Insulin/Aβ42, tau, Ng, Cerebral perfusion, Cerebral 11C-AcAc uptake, Executive function, attention, verbal abilities, emory, Capillary BHB	Positive
(Siegmann et al. 2019)	Prediabetic adults and diabetes	Participants	Clinical	Nutritional ketosis	PSQI	Weight, fasting blood glucose, HbA1c, HOMA-IR, BHB and hsCRP	Positive
(Nagpal et al. 2019)	Not	Participants	Clinical	MMKD vs. AHAD	Not mentioned	Aβ42, Aβ40, total tau, tau-p181	Positive
(Xu et al. 2010)	Not	Aged rats	Laboratory	KD	Object recognition test, T-maze test, Inclined screen test	Blood ketone levels, BHB/Cognitive performance, T-maze test: alternation rate, time to choose arms, Object recognition test, new object exploration percent, Motor performance, Inclined-screen test, Brain cortex parameters, HIF-1a protein levels, Capillary density	Positive

## 3 Neuroprotective mechanisms of ketogenic diets

**Figure 1:**
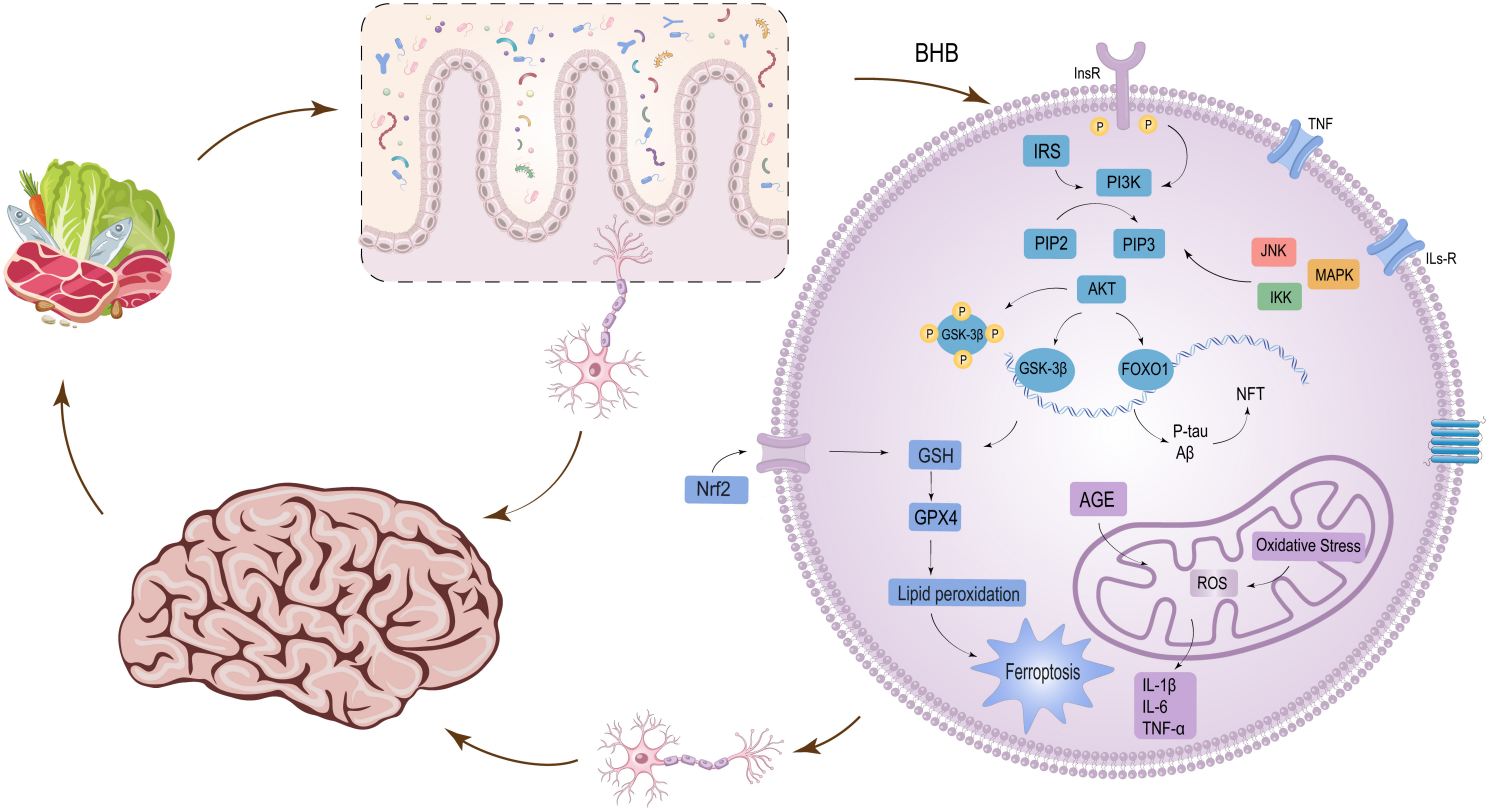
Proposed mechanisms by which low-carbohydrate ketogenic diets (LCKDs) alleviate diabetes-associated cognitive dysfunction. LCKDs elevate beta-hydroxybutyrate (BHB), which activates InsR/IRS/PI3K/AKT signaling, inhibits GSK-3β and FOXO1, and upregulates Nrf2 to reduce oxidative stress (ROS/GSH). BHB also suppresses NF-κB-driven neuro-inflammation (IL-1β, IL-6, TNF-α), decreases P-tau and Aβ, and inhibits ferroptosis via GPX4 and lipid peroxidation. Concurrently, LCKDs modulate the gut-brain axis by enriching *Akkermansia* and short-chain fatty acids, thereby attenuating systemic and central inflammation. These interconnected pathways collectively preserve cognitive function in diabetes.

The pathogenesis of diabetic cognitive dysfunction involves complex interactions between metabolic dysfunction and neurodegeneration. Key contributors include insulin resistance, cerebral microvascular damage, chronic neuroinflammation, oxidative stress, mitochondrial dysfunction, and dysregulation of the gut-brain axis (Yang et al., 2022b; Li et al., 2023; Wang et al., 2022; Little et al., 2022; Yue et al., 2024; Elzinga et al., 2025; Chen et al., 2023). Emerging evidence suggests that ketogenic diets may mitigate cognitive decline by targeting these interconnected pathways (Figure 1). This section synthesizes the evidence for these mechanisms, integrating recent findings and calibrating conclusions to the strength of the available data.

### 3.1 Amelioration of insulin resistance

Insulin resistance is a core pathophysiological feature linking T2DM to cognitive impairment. In the brain, insulin resistance disrupts signaling pathways crucial for neuronal survival, synaptic plasticity, and energy homeostasis. Notably, insulin-degrading enzyme (IDE) is responsible for cleaving both insulin and amyloid-beta (Aβ); hyperinsulinemia can competitively inhibit Aβ clearance (Kellar and Craft, 2020). The phosphatidylinositol-3 kinase/protein kinase B (PI3K/Akt) pathway, a key downstream insulin signaling cascade, is implicated in both diabetes and cognitive function (Constantino et al., 2018).

Preclinical studies indicate that ketogenic diets may modulate these pathways. In a rodent model of bipolar disorder, a ketogenic diet modulated the PI3K/Akt/HIF-1α pathway, coinciding with improved insulin sensitivity and cognitive performance (Campbell and Campbell, 2020). Short-term carbohydrate restriction in patients with MCI has been associated with reduced insulin resistance and neuroinflammation, alongside memory improvements (Gibas, 2017; Carneiro and Leloup, 2020). Mechanistically, studies in epileptic rats suggest that ketogenic diets may enhance cognitive function by modulating glutamate receptor expression and suppressing excessive MAPK pathway activation (Wang et al., 2021). Recent evidence further demonstrates that BHB directly inhibits class I histone deacetylases (HDACs), thereby upregulating genes associated with oxidative stress resistance and potentially influencing insulin sensitivity at the transcriptional level (Shimazu et al., 2013; Pali et al., 2025). While these findings are promising, direct evidence within diabetic populations remains limited and warrants further investigation.

### 3.2 Modulation of brain structure, metabolism, and perfusion

Cognitive dysfunction is underpinned by structural and functional cerebral alterations, including neuronal loss, impaired synaptic plasticity, and reduced cerebral blood flow (Liu Q. et al., 2025). Ketone bodies, particularly BHB, serve not only as an efficient brain fuel but also as signaling molecules that may influence these processes.

In aged mice, a ketogenic diet modulated the cortical synaptic proteome via the PKA signaling pathway, enhancing synaptic organization and improving memory (Acuña-Catalán et al., 2024). In APP/PS1 mice, a model of AD, a ketogenic diet restored hippocampal long-term potentiation (LTP)-a cellular correlate of learning and memory-through a BHB-mediated mechanism involving enhanced synaptic plasticity, reduced microglial inflammation, and increased brain-derived neurotrophic factor (BDNF) (Di Lucente et al., 2024). Ketogenic diets have also been shown to upregulate BDNF in the mouse cerebral cortex and reduce Aβ deposition and blood-brain barrier damage (Di Lucente et al., 2024; Al-Kuraishy et al., 2024; Yang et al., 2022a). In aged rats, cognitive improvement following a ketogenic diet was associated with elevated hypoxia-inducible factor-1α (HIF-1α) and subsequent cerebrovascular angiogenesis (Xu et al., 2010).

The latest clinical studies also provide evidence for the neuroprotective mechanism of LCKD. A modified Mediterranean-ketogenic diet (MMKD) has been associated with improved metabolic profiles, elevated cerebrospinal fluid Aβ42 levels, reduced tau protein, and increased whole-brain perfusion in neuroimaging studies (Neth et al., 2020). Furthermore, a 3-week intervention study with LCKD was shown to significantly increase plasma BHB levels, raise cerebral blood flow by 22%, and elevate plasma BDNF levels by 47% in healthy subjects (Luong et al., 2025a). These findings indicate that LCKD may serve as a potential approach for preventing and managing cognitive decline. These findings collectively suggest that ketogenic diets may exert neuroprotective effects through direct modulation of brain structure, metabolism, and perfusion.

### 3.3 Attenuation of neuroinflammation and oxidative stress

Chronic neuroinflammation and oxidative stress are pivotal in the pathogenesis of diabetic cognitive dysfunction. Hyperglycemia promotes oxidative stress and the accumulation of advanced glycation end products (AGEs), contributing to neurovascular injury (Michailidis et al., 2022). Microglia, the brain's resident immune cells, can become chronically activated, shifting to a pro-inflammatory (M1) phenotype and releasing cytokines such as interleukin-1β (IL-1β) and tumor necrosis factor-α (TNF-α), which exacerbate neuronal damage (Hou et al., 2025; Adamu et al., 2024).

Ketone bodies exhibit anti-inflammatory and antioxidant properties. In a mouse model of ischemic stroke, administration of BHB improved cognitive performance post-stroke, an effect attributed to enhanced neuronal plasticity, anti-inflammatory activity, and antioxidant capacity (Gureev et al., 2024). A 3-month ketogenic intervention in APP/PS1 mice reduced cerebral Aβ deposition and decreased pro-inflammatory cytokine levels, correlating with improved learning and memory (Jiang et al., 2023). Mechanistically, these benefits were linked to suppression of the nuclear factor-κB (NF-κB) inflammatory pathway and concurrent activation of the nuclear factor erythroid 2-related factor 2/heme oxygenase-1 (Nrf2/HO-1) antioxidant axis (Shippy et al., 2025). Recent evidence extends these findings, demonstrating that BHB suppresses NLRP3 inflammasome activation in microglia, thereby attenuating neuroinflammatory responses (Shippy et al., 2020). While compelling, these findings require replication in models of diabetic cognitive dysfunction specifically.

### 3.4 Restoration of mitochondrial function

Mitochondrial dysfunction is a hallmark of both diabetes and neurodegenerative diseases. Impaired mitochondrial bioenergetics and defective mitophagy-the selective clearance of damaged mitochondria-contribute to neuronal stress and death (Carvalho and Moreira, 2023; Shafiee et al., 2025). In the BTBR mouse model of neurodevelopmental disorders, a 2-week ketogenic diet improved mitochondrial function and ameliorated behavioral and cognitive deficits (Ahn et al., 2020). The neuroprotective effects are hypothesized to stem from ketosis-induced enhancement of mitochondrial efficiency, reduced oxidative stress, and modulation of inflammatory pathways (Pawłowska et al., 2025; Paoli et al., 2024; Jang et al., 2023).

Recent advances have elucidated more specific mechanisms. Ketone bodies have been shown to enhance mitochondrial respiration and reduce reactive oxygen species (ROS) production by increasing the oxidized-to-reduced coenzyme Q (CoQ) ratio (Sato et al., 1995; Veech et al., 2001; Puchalska and Crawford, 2017). Additionally, BHB promotes mitochondrial biogenesis via activation of the PGC-1α pathway (Gómora-García et al., 2023; Hasan-Olive et al., 2019; Nesterova et al., 2024). A recent study in aged mice demonstrated that intermittent ketogenic feeding preserved mitochondrial function and neuromuscular junction integrity, correlating with improved cognitive and physical performance (Rutkowsky et al., 2024; Wallace et al., 2021; Roslund et al., 2025). However, direct evidence linking ketogenic diet-induced changes in mitochondrial function to cognitive outcomes in diabetes-specific models remains an important research priority.

### 3.5 Regulation of the gut-brain axis

The gut microbiota and its metabolites are increasingly recognized as modulators of brain function via the gut-brain axis. In older adults with MCI, a MMKD intervention altered gut microbiota composition, increasing the abundance of specific taxa (e.g., *Enterobacteriaceae, Akkermansia*) and increasing fecal short-chain fatty acid (propionate, butyrate) levels (Nagpal et al., 2019). These changes correlated with reduced AD pathological markers in cerebrospinal fluid and improved cognition. In MCI patients, a ketogenic diet reduced levels of GABA-producing gut microbes and fecal GABA, while increasing GABA-lowering taxa, suggesting modulation of GABA signaling as a potential mechanism for cognitive improvement (Dilmore et al., 2023).

More recent research has identified that ketogenic diets increase the Akkermansia muciniphila abundance, which is associated with improved gut barrier function and reduced systemic inflammation (Tang et al., 2025; Chelakkot et al., 2018; Paone et al., 2026). The resulting reduction in peripheral inflammatory cytokines may subsequently decrease neuroinflammation via the vagus nerve and circumventricular organs (Hosoi et al., 2002; Dantzer et al., 2000). These findings highlight the gut-brain axis as a promising therapeutic target, though the specific contribution of these microbial shifts to cognitive outcomes in diabetic populations requires further exploration.

### 3.6 Inhibition of ferroptosis

Ferroptosis, an iron-dependent form of regulated cell death driven by lipid peroxidation, has emerged as a potential mechanism in neurodegenerative diseases (Ursini and Maiorino, 2020; Xu et al., 2024; Liu et al., 2024; Wang et al., 2025; Kazmirczak et al., 2024; Chen et al., 2022a; Liu and Wang, 2022). Its relevance to cognitive dysfunction is supported by studies showing that inhibiting ferroptosis alleviates cognitive deficits in rodent models of ischemia-reperfusion injury and AD (Wang et al., 2023; Naderi et al., 2025; Sousa et al., 2023; Chen et al., 2022b; Fu et al., 2022; Lu et al., 2025; Li et al., 2025). A recent study reported that a low-carbohydrate diet suppressed ferroptosis, activated the hippocampal Sirt1-Nrf2 axis, and ameliorated sleep deprivation-induced cognitive dysfunction and AD-like pathology in mice (Yang et al., 2022a). In APP/PS1 mice, a 12-week low-carbohydrate diet intervention improved cognitive function, an effect potentially mediated by BHB-induced Nrf2 activation and subsequent regulation of neuronal ferroptosis and Aβ accumulation (Qin et al., 2023).

Collectively, these convergent mechanisms position ketone bodies as pleiotropic metabolites restoring cellular homeostasis under metabolic stress. In diabetes, where chronic hyperglycemia disrupts multiple adaptive pathways, nutritional ketosis recalibrates redox balance, energy availability, and inflammatory tone-systems-level determinants of neuronal resilience. This mechanistic breadth supports ketogenic metabolic therapy as a disease-modifying strategy warranting prioritized investigation in diabetic populations at risk for cognitive decline.

## 4 Safety and clinical considerations in diabetes

Implementing ketogenic diets in individuals with diabetes requires rigorous safety monitoring due to potential interactions with glucose-lowering medications and the presence of underlying comorbidities. Short-term adverse effects commonly include gastrointestinal disturbances and electrolyte imbalances collectively termed “keto flu” (Dyńka et al., 2026; Bostock et al., 2020; Sáenz de Pipaón et al., 2020). Long-term risks, however, necessitate more nuanced consideration in this population.

### 4.1 Medication interactions

Concomitant use of insulin or insulin secretagogues (e.g., sulfonylureas) with carbohydrate restriction significantly elevates hypoglycemia risk, mandating dose reductions upon diet initiation and continued adjustment throughout the intervention. The combination with sodium-glucose cotransporter-2 inhibitors warrants particular caution due to an increased, albeit rare, risk of euglycemic diabetic ketoacidosis (Long et al., 2021; Peters et al., 2015; Nasa et al., 2021). Recent evidence from a long-term case report demonstrated a 43% reduction in daily insulin requirements with ketogenic diet implementation, underscoring the necessity for substantial dose adjustments upon diet initiation (Koutnik et al., 2024; Gardemann et al., 2023; Wolver et al., 2021). Clinical practice guidelines emphasize the importance of close glucose monitoring and individualized insulin modification to mitigate hypoglycemia risk.

### 4.2 Dyslipidemia

Ketogenic diets high in saturated fat may elevate low-density lipoprotein cholesterol in susceptible individuals termed “hyper-responders,” potentially exacerbating cardiovascular risk (Norwitz et al., 2021; Budoff et al., 2024). Lipid profiles should be monitored at baseline and periodically during intervention (Kirkpatrick et al., 2019), with emphasis on dietary fat quality prioritizing monounsaturated and polyunsaturated fats over saturated fats (Liu Y. et al., 2025).

### 4.3 Renal considerations

High dietary protein intake, common in some low-carbohydrate ketogenic diet versions, may increase glomerular pressure and warrants caution in patients with chronic kidney disease (de Lorenzo et al., 2024). Nephrolithiasis represents a recognized risk, particularly attributable to urinary pH changes and hypocitraturia, with adequate hydration and potassium citrate supplementation potentially mitigating this risk (Ko et al., 2020).

### 4.4 Nutrient deficiencies

Long-term carbohydrate restriction may precipitate deficiencies in vitamins (e.g., B vitamins, vitamin D), minerals (e.g., magnesium, selenium), and fiber, necessitating supplementation or structured dietary planning with a registered dietitian (Churuangsuk et al., 2019; Tay et al., 2020).

### 4.5 Contraindications

Absolute contraindications include pregnancy, lactation, eating disorders, pancreatitis, liver failure, and specific metabolic disorders such as carnitine deficiency, porphyria, and pyruvate carboxylase deficiency (Tay et al., 2020; Barrea et al., 2024). Relative contraindications require careful risk-benefit assessment on an individual basis. These safety considerations collectively underscore the necessity of multidisciplinary management when implementing ketogenic interventions in diabetic populations.

## 5 Limitations and future directions

This review synthesizes current evidence positioning ketogenic metabolic therapy as a potential neuroprotective strategy for diabetes-associated cognitive dysfunction, yet several limitations warrant acknowledgment. The existing literature remains predominantly preclinical, with human studies confined to small samples, short durations, and heterogeneous interventions that impede cross-study comparison. Most clinical evidence derives from mild cognitive impairment or Alzheimer's disease populations rather than diabetic cohorts, limiting direct translatability. Furthermore, the absence of standardized cognitive endpoints and inconsistent reporting of diabetes- relevant parameters-such as hypoglycemia history, microvascular burden, and neuroimaging markers-precludes definitive conclusions regarding efficacy.

Moreover, the ε4 allele of apolipoprotein E (APOE4) represents the strongest genetic risk factor for Alzheimer's disease (AD), conferring an approximately fourfold increase in AD risk among ~34 million American women and 75 million European women. Metabolic deficits in the brain are detectable in APOE4 carriers decades before clinical symptom onset (Di Lucente et al., 2026). The Alzheimer's Disease Assessment Scale-Cognitive Subscale (ADAS-Cog) is widely regarded as the gold standard for assessing cognitive impairment in AD. Interventions based on ketogenic diets (KD) or medium-chain triglycerides (MCT) have been associated with improvements in ADAS-Cog scores, particularly in individuals who are APOE4 non-carriers (Taylor et al., 2017; Reger et al., 2004). Specifically, MCT supplementation has been linked to improved ADAS-Cog performance in APOE4-negative individuals, whereas no statistically significant cognitive benefits have been observed among APOE4-positive participants (Reger et al., 2004). In a multicenter, randomized, placebo-controlled trial, supplementation with AC-1202 led to significant improvements in ADAS-Cog scores in individuals who were APOE4-negative with mild-to-moderate Alzheimer's disease, while no significant benefit was detected in those carrying the APOE4 allele (Henderson et al., 2009). Collectively, these findings highlight the critical role of APOE4 genotype in modulating the therapeutic response to ketogenic interventions and underscore the need for genotype-informed, personalized treatment strategies.

Several priorities emerge for future investigation. First, rigorous randomized controlled trials are needed in phenotypically characterized diabetic populations, incorporating standardized cognitive batteries alongside concurrent assessment of glycemic control, vascular status, and brain imaging biomarkers (Luong et al., 2025b; Dyńka et al., 2026). Second, intervention protocols require harmonization regarding carbohydrate thresholds, dietary fat quality, ketosis monitoring, and duration to enable cross-study comparability. Third, mechanistic studies integrated within clinical trials should confirm target engagement-via neuroimaging of brain ketone uptake or cerebrospinal fluid analysis of neurodegenerative and inflammatory markers-and identify response biomarkers to guide patient stratification. Fourth, long-term safety data beyond 12 months are essential, particularly regarding cardiovascular outcomes, renal function, and medication interactions in diabetic patients with varying comorbidities.

Addressing these priorities will determine whether ketogenic metabolic therapy can transition from mechanistic promise to evidence-based clinical application for mitigating cognitive decline in the aging diabetic population.

## 6 Conclusions and future perspectives

Current evidence suggests ketogenic diets exert neuroprotection against diabetes-associated cognitive dysfunction through synergistic mechanisms-improved insulin sensitivity, reduced neuro-inflammation, enhanced mitochondrial function, and gut-brain axis modulation. Substantial gaps remain, however. The evidence base is predominantly preclinical; human studies are limited by small sample sizes, short durations, and heterogeneous interventions. Inconsistent outcome reporting and insufficient attention to diabetes-specific safety further limit translational value.

Advancing the field requires: (i) rigorous randomized trials in diabetic populations using standardized cognitive endpoints; (ii) harmonized intervention protocols defining carbohydrate thresholds, fat quality, and ketosis monitoring; (iii) evidence-based safety guidelines tailored to diabetes, including long-term data beyond 12 months; and (iv) mechanistic studies integrated within trials to confirm target engagement and identify response biomarkers.

Addressing these priorities is essential to determine whether ketogenic metabolic therapy represents a safe, effective strategy to mitigate cognitive decline in the aging diabetic population.
